# Editorial Department

**Published:** 1872-10

**Authors:** 


### EDITORIAL DEPARTMENT.

We call attention to our Editorial Department. We do not desire to monopolize our editorial space, consequently, we have invited our friends to express their views, upon all legitimate topics, in it. We desire they should avail themselves of this for the general good of the profession, the necessities and wants of which our brethren can so fully appreciate and feel. We will cull from all letters such extracts as may be deemed of special importance in promoting the interests of the profession. We, therefore, earnestly invite our friends everywhere to write short paragraphs covering some principle for the common good. In this way we can give our readers, monthly, many useful hints, many valuable thoughts for their benefit and the good of the sincere, earnest practitioner. Will our friends not bear this in mind and agree to work for the honor and glory of a common cause? We think they will. Friends, send us your thoughts. They will do good—and good only.

